# The impact of chitooligosaccharides with a certain degree of polymerization on diabetic nephropathic mice and high glucose‐damaged HK‐2 cells

**DOI:** 10.1002/fsn3.4078

**Published:** 2024-03-22

**Authors:** Yuwen Liu, Ran Zhang, Jiaqi Zou, Hao Yin, Mengyao Zhao, Liming Zhao

**Affiliations:** ^1^ State Key Laboratory of Bioreactor Engineering, R&D Center of Separation and Extraction Technology in Fermentation Industry, School of Biotechnology East China University of Science and Technology Shanghai China; ^2^ Shanghai Frontiers Science Center of Optogenetic Techniques for Cell Metabolism Shanghai China; ^3^ Organ Transplant Center Shanghai Changzheng Hospital Shanghai China

**Keywords:** chitooligosaccharides, diabetic nephropathy, Nrf2/Keap1 pathway, oxidative stress

## Abstract

Diabetic nephropathy (DN) is a primary diabetic complication ascribed to the pathological changes in renal microvessels. This study investigated the nuclear factor erythroid 2‐related factor 2 (Nrf2)/Kelch ECH associating protein (Keap1)/antioxidant response element (ARE) signaling pathway impact of chitooligosaccharides (COS) with a certain degree of polymerization (DP) on DN mouse models and high glucose‐damaged human kidney 2 (HK‐2) cells. The findings indicated that COS effectively reduced the renal function indexes (uric acid [UA], urinary albumin excretion rate [UAER], urine albumin‐to‐creatinine ratio [UACR], blood urea nitrogen [BUN], and creatinine [Cre]) of DN mice. It increased (*p* < .05) the superoxide dismutase (SOD), glutathione peroxidase (GSH‐Px), and catalase (CAT) antioxidant enzyme activity in the serum and kidneys, and decreased (*p* < .05) the malondialdehyde (MDA) content. The mechanistic investigation showed that COS significantly increased (*p* < .05) *Nrf2* and downstream target gene (*GCLM*, *GCLC*, *HO‐1*, and *NQO‐1*) expression, and substantially decreased (*p* < .05) *Keap1* expression. The protein level was consistent with the messenger RNA (mRNA) level in in vitro and in vivo models. The docking data indicated that COS and Keap1 protein binding included six hydrogen bond formation processes (Gly364, Arg415, Arg483, His436, Ser431, and Arg380). The COS intervention mechanism may be related to the Nrf2/Keap1/ARE antioxidant pathway. Therefore, it provides a scientific basis for COS application in developing special medical food for DN patients.

## Graphical abstract

The nuclear factor erythroid 2‐related factor 2/Kelch ECH associating protein (Keap1)/antioxidant response element signaling pathway impact chitooligosaccharides with a certain degree of polymerization (DP) on diabetic nephropathy mouse models and high glucose‐damaged HK‐2 cells.

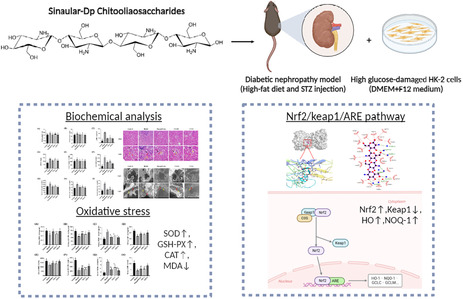

## 1 INTRODUCTION

Economic improvements and changes in dietary patterns have increased the prevalence of chronic diseases, such as diabetes mellitus (DM). In 2021, the International Diabetes Federation (IDF) reported that of the 537 million (10.5%) global diabetes cases, 141 million were identified in China, with a prevalence rate of 11.6% (Edward et al., 2021). DM primarily consists of insulin‐dependent type 1 DM (T1DM) and non‐insulin‐dependent type 2 DM (T2DM). Blood sugar levels continuously rise in T2DM patients due to the metabolic dysfunction of sugar and are caused by absolute or relative insulin sensitivity decrease, which involves a number of target organs. Therefore, failing to control diabetes progression leads to complications (American Diabetes Association, 2009).

Diabetic nephropathy (DN) involves diabetes‐induced nerve damage and is the primary cause of end‐stage renal disease (Atkins, 2005), showing a prevalence rate as high as 40% in those with T2DM (Maggiore et al., 2017). Although the pathogenesis of DN is unclear, several studies have shown that high blood sugar causes oxidative stress (OS) and activates inflammatory factors, which may be the main reason for its occurrence (Butkowski & Jelinek, 2016; Kopel et al., 2019; Vaziri & Rodríguez‐Iturbe, 2006; Wellen & Hotamisligil, 2005). No drugs are currently available that directly target DN and clinical symptoms are mainly alleviated via hypoglycemic and hypertensive methods (Han & Sun, 2015).

Chitooligosaccharides (COS) (degree of polymerization 2–10) are derived from D‐glucosamine, or a small quantity of N‐acetyl‐D‐glucosamine, connected by β‐1,4‐glucoside bonds via enzymatic chitosan hydrolysis or chemical degradation (Zhao, 2019). COS exhibits cationic characteristics, good water solubility, a low molecular weight, and low viscosity, presenting physiological activities, such as glucose and lipid metabolism regulation, antioxidant properties, and liver protection (Ji et al., 2021; Wei et al., 2012; Xie et al., 2016; Zhao et al., 2019). Previous studies have shown that COS effectively ameliorates glucose tolerance and reduces blood lipid and glucose levels in diabetic animal models. Moreover, COS reduces insulin resistance (IR) by increasing glycogen synthesis and insulin signal transduction and inhibiting liver and muscle gluconeogenesis. It also increases insulin secretion by protecting and restoring islet cell function (You et al., 2022). Therefore, although COS performs well in the diabetes model, its ability to improve DN requires further investigation.

This study attempts to clarify the COS antioxidant activity with a specific degree of polymerization (DP). It assesses the therapeutic effect of COS on DN using in vivo and in vitro models and antioxidant targets. In addition, it illustrates the molecular mechanism underlying the hypoglycemic impact of specific singular‐DP COS using structural model calculations and docking to perform computational molecular simulations. The findings of this study help elucidate the association between the activity and chemical structures of functional oligosaccharides, providing a foundation for bioactive anti‐DN drug development.

## 2 METHODS AND MATERIALS

### 2.1 The COS preparation

An enzyme‐membrane coupling reactor system was employed for chitosan degradation to prepare the COS according to a method delineated in previous studies with some modifications (Qin et al., 2018). The final COS (molecular weight 662.64 g/mol) product was quantified and characterized via mass spectrometry (MS) and a high‐performance liquid chromatography (HPLC) system, as shown in Figures S1 and S2.

### 2.2 Animal experiments

The experimental protocol adhered to the ARRIVE (Animal Research: Reporting of In Vivo Experiments) guidelines. The animal experiments and treatments were carried out in strict compliance with the National Research Council's Guide for the Care and Use of Laboratory Animals, and were approved by The East China University of Science and Technology Bioethics Committee (No: ECUST‐2022‐084; Shanghai, China). C57BL/6J mice (male; 6 weeks old; specific pathogen‐free [SPF]) were supplied by Shanghai JieSiJie Laboratory Animal Co., Ltd. in Shanghai, China.

#### 2.2.1 Treatment design

**FIGURE 1:**
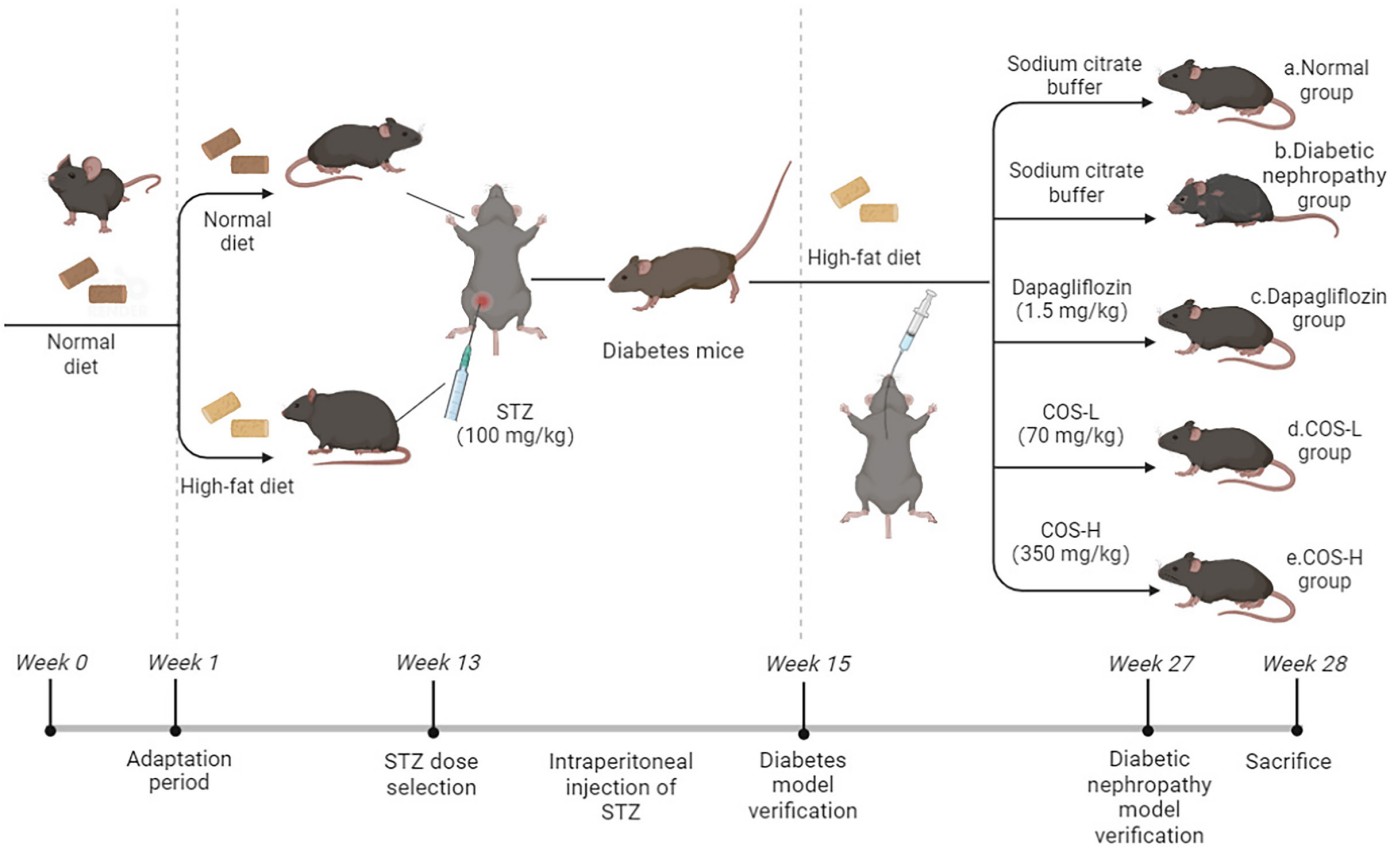
Animal grouping diagram.

Figure 1 shows the grouping. Ten C57BL/6J mice received a normal diet and were labeled group a (Normal group). The remaining mice were given a 10‐week 60% high‐fat diet. At week 11, the animals were intraperitoneally injected with 100 mg/kg streptozotocin (STZ), and their blood sugar was monitored for 2 weeks. The random blood sugar value exceeded 16.5 mol/L, showing the successful establishment of the diabetes model. The diabetic mice were separated into five random groups (10/group; group b–group e) and fed a high‐fat diet for another 12 weeks. Five of the 10 animals from each group were selected for biochemical experiment and pathological assay.

The daily intragastric administration included (a) sodium citrate buffer (Normal group), (b) sodium citrate buffer (DN group), (c) 1.5 mg/kg dapagliflozin treatment (commonly used clinical therapeutic drugs), (d) low‐dose COS at 70 mg/kg, and (e) high‐dose COS at 350 mg/kg.

### 2.3 Biochemical index

After 12 weeks of administration, a urinary microalbumin immunosorbent assay (Jining Shiye, Shanghai, China) was used to determine the urinary microalbumin content. Nine times the volume of normal saline was added a tissue weight (g) to volume (mL) = 1:9 ratio to obtain a 10% homogenate, which was subjected to ice‐water bath mechanical homogenization and centrifugation at 4000*g* for 10 min. Creatinine, urea nitrogen, and uric acid kits (Nanjing Jiancheng, China) were used to measure the corresponding content in the blood and tissue.

### 2.4 Pathological assay

Hematoxylin–eosin (HE) staining: The kidney tissues of each experimental group were fixed using a 4% paraformaldehyde solution, followed by graded ethanol dehydration. The samples were placed in paraffin, serially sectioned at 4.0 μm, and subjected to standard HE staining. The histopathological image was evaluated and recorded using a light microscope (Nikon Eclipse Si, NIKON, Japan).

Transmission electron microscopy (TME): Osmic acid and glutaraldehyde were used to fix a small sample piece, followed by acetone gradient dehydration. The sample piece was embedded in EMBed 812 epoxy resin and sectioned into ultramicrocuts using a Leica UC6 ultramicrotome (Leica). After lead citrate staining, the sections were observed via TME using a HT7800/HT7700 transmission electron microscope (Hitachi, Japan).

### 2.5 Oxidative stress (OS) index detection

The mice were fasted overnight at 12 weeks of administration. Blood was collected from the orbital venous plexus and centrifuged for 15 min at 106*g* to separate the serum. The 10% homogenate was prepared according to the tissue volume ratio. CAT, GSH‐Px, MDA, and SOD kits (Nanjing Jiancheng, China) were used to determine the corresponding content and enzyme activity in the blood and kidney tissue.

### 2.6 Cell culture and processing

Ham's F‐12 Nutrient Mix (F‐12) (Gibco, USA) containing a 1% penicillin–streptomycin solution and Dulbecco's Modified Eagle's Medium (Gibco, USA) were used for HK‐2 cell culturing in a 37°C, 5% carbon dioxide (CO_2_) cell incubator. The cells proliferating well during the logarithmic growth phase were selected for further examination. The cultured HK‐2 cells were separated into three treatment groups: the Control group treated with normal culture medium, the Model group treated with glucose for 48 h, and the Intervention group treated with COS for 48 h.

### 2.7 Real‐time fluorescence quantitative PCR (qPCR)

An Animal Total RNA Isolation Kit (Vazyme, China) was used according to the instructions of the manufacturer to isolate the RNA, after which an RT Reagent Kit (Vazyme, China) was employed to synthesize complementary DNA (cDNA) at 37°C for 15 min. Next, the reverse transcriptase was inactivated at 85°C for 5 s. The polymerase chain reaction (PCR) parameters included pre‐denaturation for 30 s at 95°C for 30 s, 40 cycles of denaturation for 5 s at 95°C, and annealing for 30 s at 55°C. The target quantity was determined via comparative cycle threshold Ct, normalized to an endogenous glyceraldehyde 3‐phosphate dehydrogenase reference, and relative to a calibrator (2^−△△ct^). The RT‐qPCR experiments were repeated three times. Tables 1 and 2 list the primers used.

**TABLE 1:** The mouse primer sequences used during qPCR.

Genes	Forward primers (5′‐3′)	Reverse primers (5′‐3′)
Nrf2	CTTTAGTCAGCGACAGAAGGAC	AGGCATCTTGTTTGGGAATGTG
Keap1	GAAGAGGCGGCAGAAGAAG	GCTCCAGGGCTATGACAGAT
NQO‐1	AGCTGGAAGCTGCAGACCTG	CCTTTCAGAATGGCTGGCA
HO‐1	GGTGATGGCTTCCTTGTACC	AGTGAGGCCCATACCAGAAG
GCLM	TGACTCACAATGACCCGAAA	GATGCTTTCTTGAAGAGCTTCCT
GCLC	ATCTGCAAAGGCGGCAAC	ACTCCTCTGCAGCTGGCTC
GAPDH	AGAAGGCTGGGGCTCATTTG	AGGGGCCATCCACAGTCTTC

**TABLE 2:** The human primer sequences used during qPCR.

Gene	Forward primers (5′‐3′)	Reverse primers (5′‐3′)
Nrf2	ACACGGTCCACAGCTCATC	TGTCAATCAAATCCATGTCCTG
Keap1	GGAGGCTATGATGGTCACAC	AGTTCTGCTGGTCAATCTGC
NQO‐1	CGCAGACCTTGTGATATTCCAG	CGTTTCTTCCATCCTTCCAGG
HO‐1	CAGTCAGGCAGAGGGTGATA	TGAGTGTAAGGACCCATCGG
GCLM	AATCTTGCCTCCTGCTGTGTGA	TGCGCTTGAATGTCAGGAATGC
GCLC	AACCCAGAGGAAGGAGGCGCATC	GTCTGGCTGAGAAGCCTTTGATGC
GAPDH	CAATGACCCCTTCATTGACC	TTGATTTTGGAGGGATCTCG

### 2.8 Western blotting

Radioimmunoprecipitation assay (RIPA) lysate (containing 100× phosphatase inhibitor and 100× protease inhibitor) was added to 100 mg of fresh animal tissue and processed in an ice bath grinder at 60 Hz for 120 s. The ice was cracked at 12,000 *g* for 30 min and centrifuged for 10 min, after which the clarified solution was absorbed. A bicinchoninic acid (BCA) kit was employed to ascertain the protein concentrations of the respective groups, which were normalized using double‐distilled water (ddH_2_O), after which the protein samples were prepared by adding 5× loading buffer and subjected to sodium dodecyl sulfate‐polyacrylamide gel electrophoresis (SDS‐PAGE). Next, the samples were placed on a polyvinylidene fluoride (PVDF) membrane using the sandwich method, where they were enclosed with skim milk powder for 1 h, followed by a TBST (Tris‐buffered saline with 0.1% Tween 20) washing process that was repeated three times. The primary antibody was incubated overnight at 4°C, after which the TBST membrane washing process was repeated three times. Then, the secondary antibody was added at a 1:3000 dilution ratio and incubated for 1 h at room temperature, after which the membrane was washed three times with TBST. The film was placed in a luminescent imager to visualize the protein bands, while the ImageJ software was used for gray‐scale analysis.

### 2.9 Molecular docking

The plane drawings were obtained according to the molecular COS monomer formula using the ChemDraw 3D software. The three‐dimensional (3D) target protein structure was obtained from the Research Collaboratory for Structural Bioinformatics Protein Data Bank (RCSB PDB) (www.rcsb.org), while AutoDock Vina 1.1.2 was employed for semiflexible docking to determine the lowest docking score conformation for bonding mode assessment. The PyMol software was used for image optimization, while a two‐dimensional (2D) interaction diagram was drawn using LigPlot.

### 2.10 Data analysis

The mean ± SEM (standard error of the mean) was used for data expression. GraphPad Prism 8.0.1 was employed for data processing, analysis, and mapping. Statistical Product and Service Solutions (SPSS, Version 2.1), while comparisons were performed via one‐way analysis of variance and Duncans multiple range test to determine the difference significance. Values of *p* < .05 indicated significant differences, which were represented by different letters.

## 3 RESULTS

### 3.1 Validation of the DN model

**FIGURE 2:**
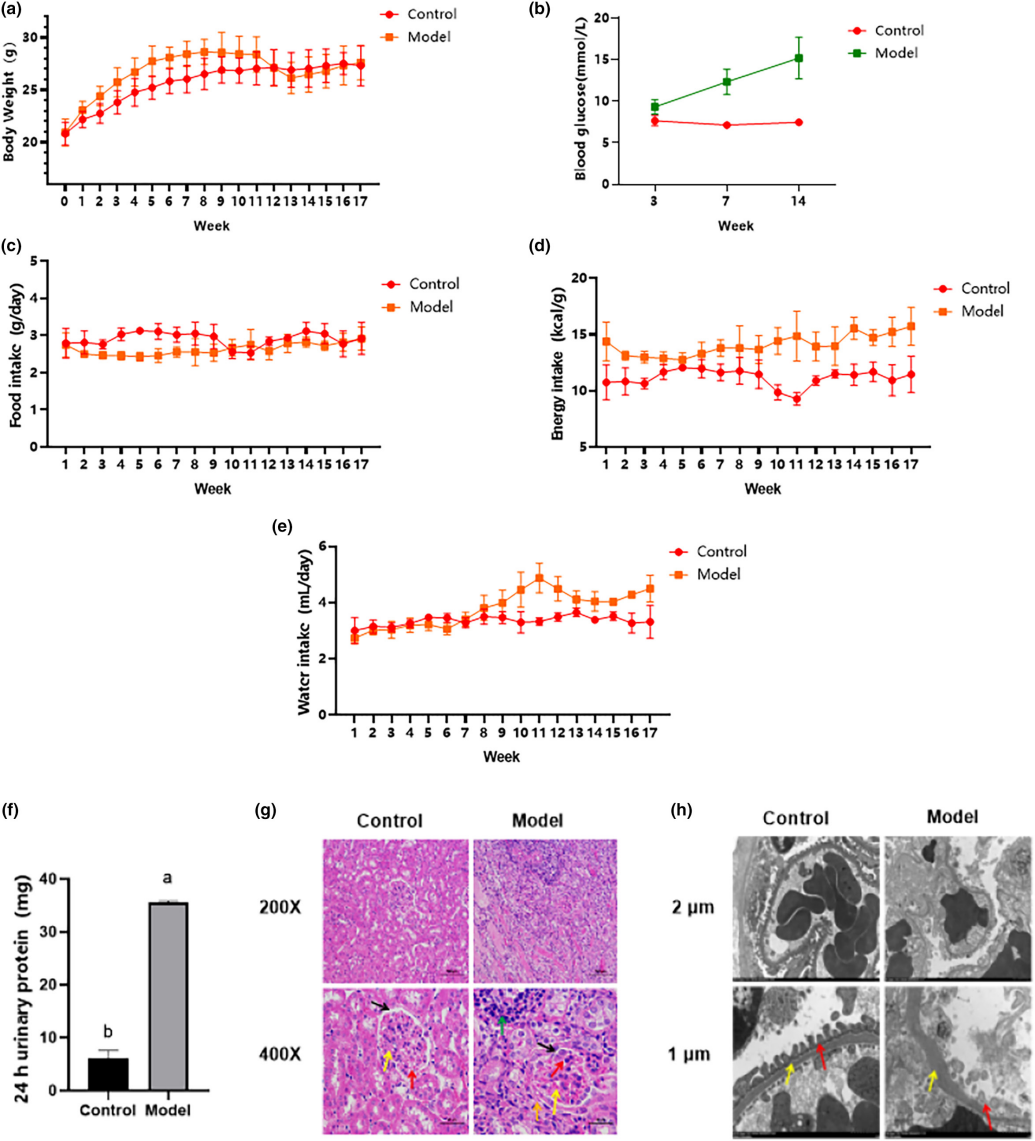
Establishment of the DN mouse model. (a) Body weights. (b) Blood glucose levels. (c) Food consumption. (d) Energy consumption. (e) Water consumption. (f) 24 h urine protein content. (g) HE staining (multiples 200× and 400×). (h) Transmission electron microscopy (multiples 2 μm and 1 μm). (a)–(f) *n* = 10, (g) and (h) *n* = 5. Significant differences (*p* < .05) are indicated by different letters.

No significant body weight differences were evident during the first week of the adaptation period. After 5 weeks of feeding, the body weights of the mice differed significantly (*p* < .05) between the model and normal groups, as shown in Figure 2a. Although the model group body weight values decreased after STZ injection, the food and water intake increased with time extension. The random blood glucose level of the model group increased significantly (*p* < .05), reaching 16.5 mmol/L (Figure 2c). Therefore, the C57BL/6J mouse diabetes model created by combining high‐sugar and ‐fat diets with STZ was successfully established.

Compared with the normal group, the urinary trace protein (Figure 2f) in the model group increased significantly (*p* < .05) after 23 weeks of feeding. Moreover, cortical HE staining (Figure 2g) indicated a significant mesangial matrix increase in the model group (shown by the yellow arrow), while narrow cavity positions (shown by black arrow) and capillaries (shown by red arrow) were evident. Inflammatory infiltrations were also visible, as shown by the green arrow, indicating that the mice in the model group had developed inflammation. Diffuse lesions were found in cells outside the glomerulus in the model group, as shown by the orange arrow. Transmission electron microscope observation (Figure 2h) revealed diffuse podocyte foot processes in the model group (indicated by the red arrow), unclear endothelial cell boundaries compared with the normal group, and a thickened basement membrane (shown by the yellow arrow). Based on the above results, it was considered that the DN model was successfully established.

### 3.2 The effect of COS on renal function and pathological changes

**FIGURE 3:**
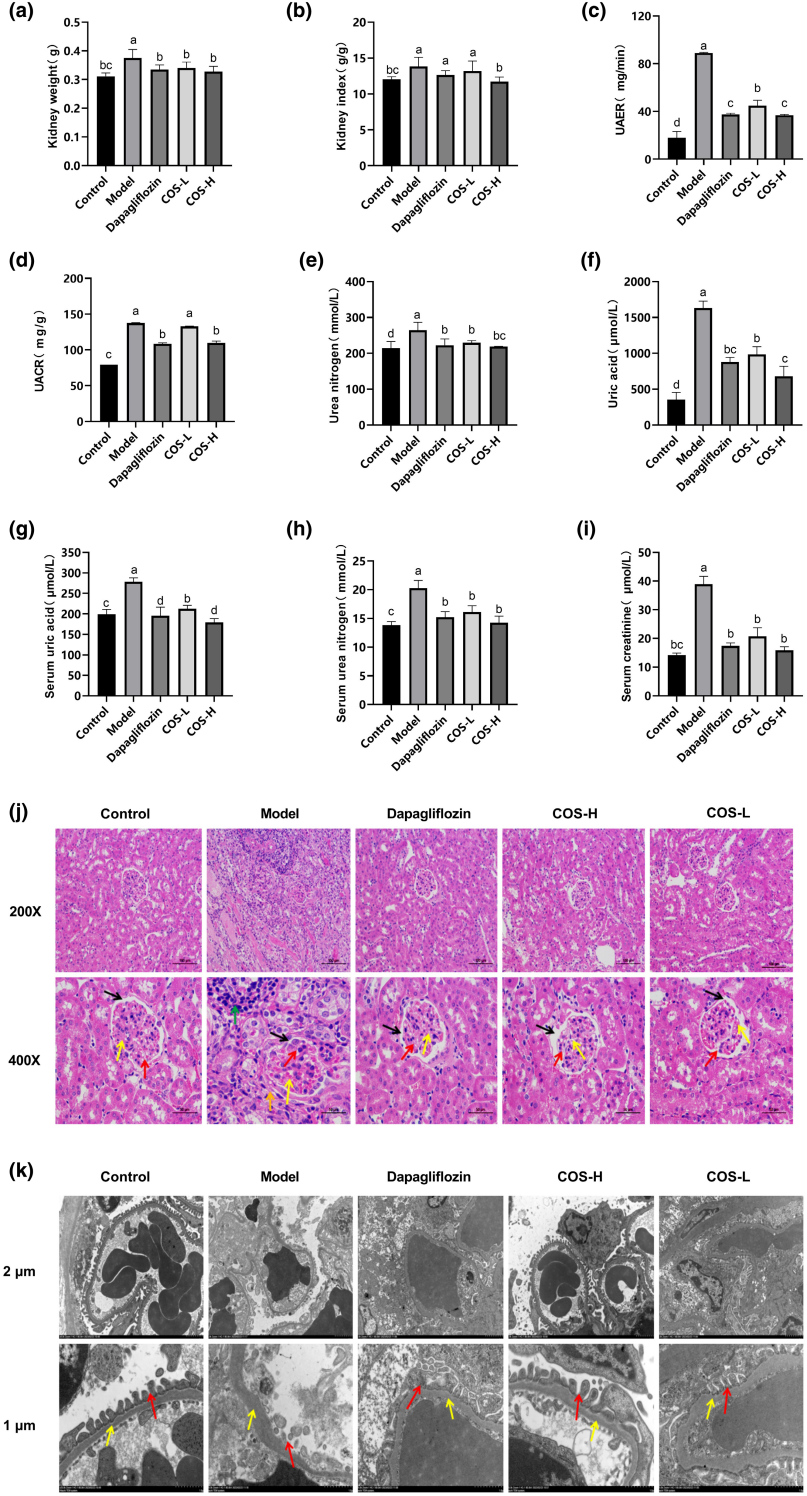
The COS impact on the renal function of the mice. Changes in (a) the total renal weight and (b) the renal hypertrophy index of mice during intervention. The (c) UAER, (d) UACR, and (e) and (f) urea nitrogen level and uric acid content in the urine. (g)–(i) The uric acid level, urea nitrogen content, and creatinine in the serum. (g) HE staining (multiples 200× and 400×). Mesangial matrix (yellow arrow), capillary width (red arrow), cavity size (black arrow), inflammatory infiltration (green arrow), and cell diffusion (orange arrow). (k) Transmission electron microscopy (multiples 2 μm and 1 μm). Podocyte foot processes (red arrow), basement membrane (yellow arrow). (a)–(i) *n* = 10, (j) and (k) *n* = 5. Significant differences (*p* < .05) are indicated by different letters.

The kidney tissues were weighed after 12 weeks of intervention. As shown in Figure 3a, the total weight of the model group kidneys differed significantly (*p* < .05) from the intervention and normal groups. The renal hypertrophy index was obtained, according to the mouse body weight to total kidney volume ratio. As shown in Figure 3b, no significant variation was apparent between the dapagliflozin and low‐dose COS groups and the model group, while the high‐dose COS group values were considerably below (*p* < .05) the model group levels. The main urine index in the model group was higher (Figure 3c–f, *p* < .05) than in the normal group. Significant differences (*p* < .05) were evident between the model, intervention, and positive control groups, indicating that disease progression was controlled in both the intervention and positive drug groups. In addition, the effect was dose‐dependent, with high‐dose COS and positive drug treatment yielding similar results. Considerably lower (*p* < .05) UA, BUN, and CRE levels were found in the serum of the intervention group than in the model group (Figure 3g–i). The high‐dose COS group serum UA was significantly lower (*p* < .05) than in the dapagliflozin group. However, no statistical differences were evident between the BUN and CRE indexes of the high‐ and low‐dose COS and positive drug groups. Therefore, since high COS doses significantly reduced the renal hypertrophy and renal injury indexes in the urine and serum, it was presumed to provide good renal protection.

Pathological analysis is the most important indicator of DN. HE staining (Figure 3j) showed that the mesangial matrixes in dapagliflozin, low‐ and high‐dose COS groups were lower than in the model group. This increased the cavity sizes, which were more pronounced in the high‐dose COS group, while the capillaries and cell morphology were clearer. TME (Figure 3k) showed basement membrane thickening in the low‐dose COS group and unclear podocyte foot processes in the dapagliflozin group. The high‐dose COS group displayed a more intact pedicular structure, clear endothelial cells, and a reduced basement membrane than the model group.

### 3.3 The COS impact on the OS in the serum and kidneys of the DN mice

**FIGURE 4:**
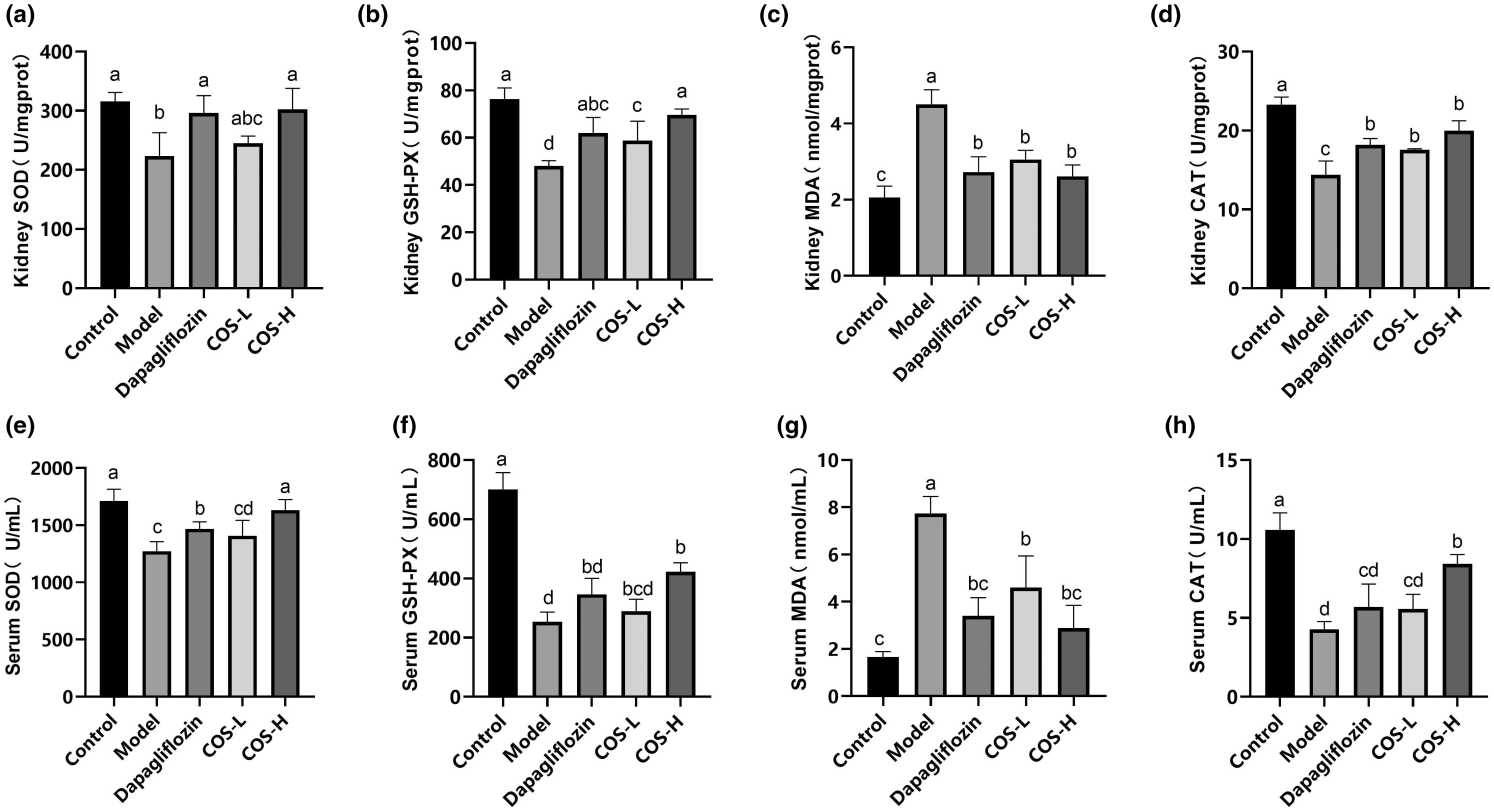
The COS intervention impact on the OS indicators. (a) The renal SOD. (b) The renal GSH‐Px. (c) The renal CAT. (d) The renal MDA. (e) The serum SOD. (f) The serum GSH‐Px. (g) The serum CAT. (h) The serum MDA. (a)–(h) *n* = 10. Significant differences (*p* < .05) are indicated by different letters.

After COS intervention for 12 weeks (Figure 4), high‐dose COS and positive drug groups displayed the same effect on the kidney tissue results, showing higher SOD, GSH‐Px, and CAT activity and lower MDA content. No significant differences were evident between the OS indexes in the serum of the model and low‐dose COS groups. The high‐dose COS group exhibited the best SOD and CAT values in the serum, with substantial differences (*p* < .05) between the COS and model groups. The results showed that a low‐COS dose reduced the MDA content, while a high dose increased the CAT, GSH‐Px, and SOD levels. Therefore, a high COS dose effectively alleviated OS.

### 3.4 The COS impact on the Nrf2/Keap1/antioxidant response element (ARE) pathway in vivo

**FIGURE 5:**
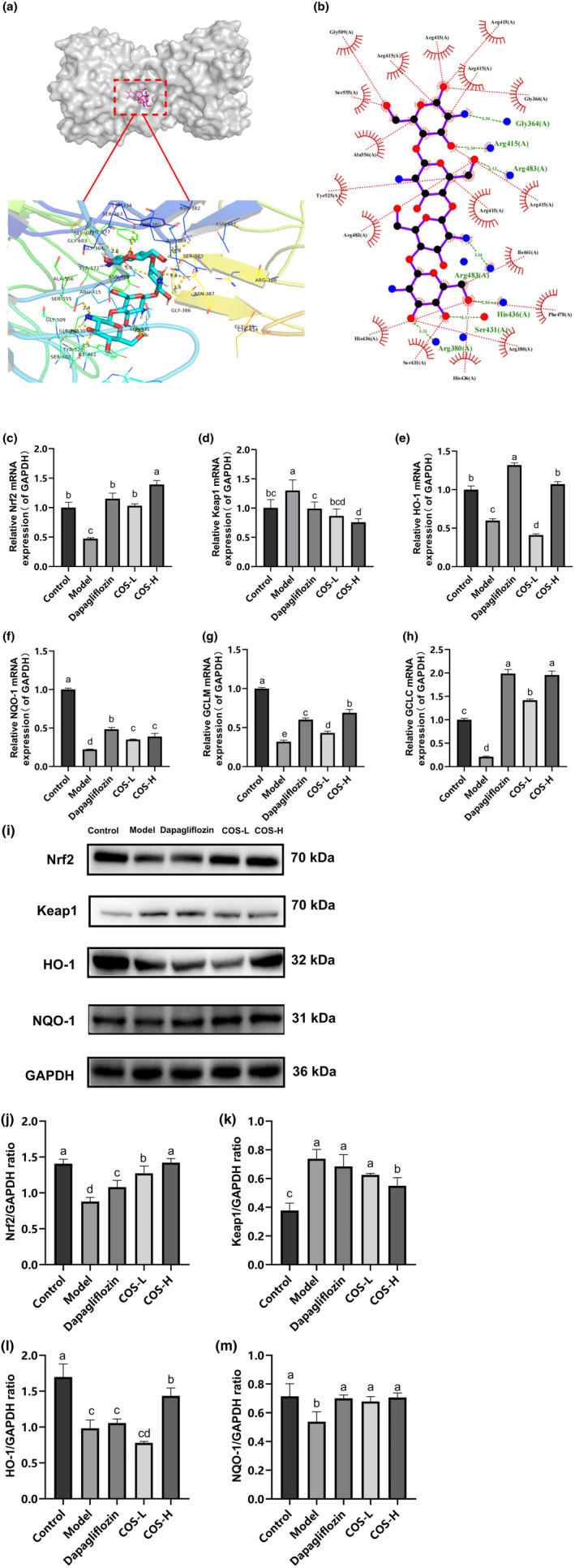
The effect of COS on the Nrf2/Keap1/ARE pathway in vivo. (a) and (b) The docking images of the Keap1 protein with COS polymerization. (c) Nrf2. (d) Keap1. (e) HO‐1. (f) NQO‐1. (g) GCLM. (h) The GCLC mRNA expression level. (i) The Western blotting representation of the related factors. (j) Nrf2. (k) Keap1. (l) HO‐1. (m) NQO‐1. (a)–(m) *n* = 3. Significant differences (*p* < .05) are indicated by different letters.

The docking results showed a COS and Keap1 protein‐binding energy of −7.4 kcal/mol, indicating that COS and Keap1 protein binding could form a stable state (Figure 5a,b). The LigPlot results revealed COS and Keap1 hydrogen bond formation with Gly364, Arg415, Arg483, His436, Ser431, and Arg380, as well as with Gly509, Arg415, Ser555, Ala556, Tyr525, Arg483, His436, Ser431, Arg380, Phe478, Ile461, Arg415, and Gly364 amino acid hydrophobicity. It is speculated that COS can bind directly to Keap1, promote Nrf2 monomer dissociation from the Nrf2–Keap1 dimer, and shift into the nucleus to upregulate downstream target gene expression.

The effect of COS on the protein and gene levels in the Nrf2/Keap1/ARE pathway was determined to elucidate the COS intervention mechanism in the in vivo DN model. As illustrated in Figure 5c–m, the expression levels of the *Nrf2*, *HO‐1*, *NQO‐1*, *GCLC*, and *GCLM* mRNA in the DN group showed a significant decline (*p* < .05), while that of *Keap1* increased. The *Nrf2* mRNA expression increased in the low‐dose COS, high‐dose COS, and positive control groups (*p* < .05). The glutamate–cysteine ligase regulatory (GCLM) and catalytic (GCLC) subunits are downstream genes of the Nrf2 transduction signal axis that can regulate glutathione (GSH) synthesis (Franklin et al., 2009). The results indicated significantly increased (*p* < .05) *HO‐1*, *GCLC*, and *GCLM* gene expression in the low‐ and high‐dose COS and positive control groups. A high COS dose increased the heme oxygenase 1 (HO‐1), NAD(P)H quinone oxidoreductase 1 (NQO‐1), glutamate–cysteine ligase (GCLC), and glutamate–cysteine ligase modified subunit messenger RNA (GCLM mRNA) expression levels more effectively. Further determination showed significantly decreased (*p* < .05) NQO‐1, HO‐1, and Nrf2 protein levels in the DN group, while those of Keap1 increased substantially (*p* < .05). These protein levels were considerably higher (*p* < .05) in the high‐dose COS group than in the model group, while those of Keap1 were substantially lower (*p* < .05), which corresponded with the gene‐level findings.

### 3.5 The effect of COS on the Nrf2/Keap1/ARE pathway in vitro

**FIGURE 6:**
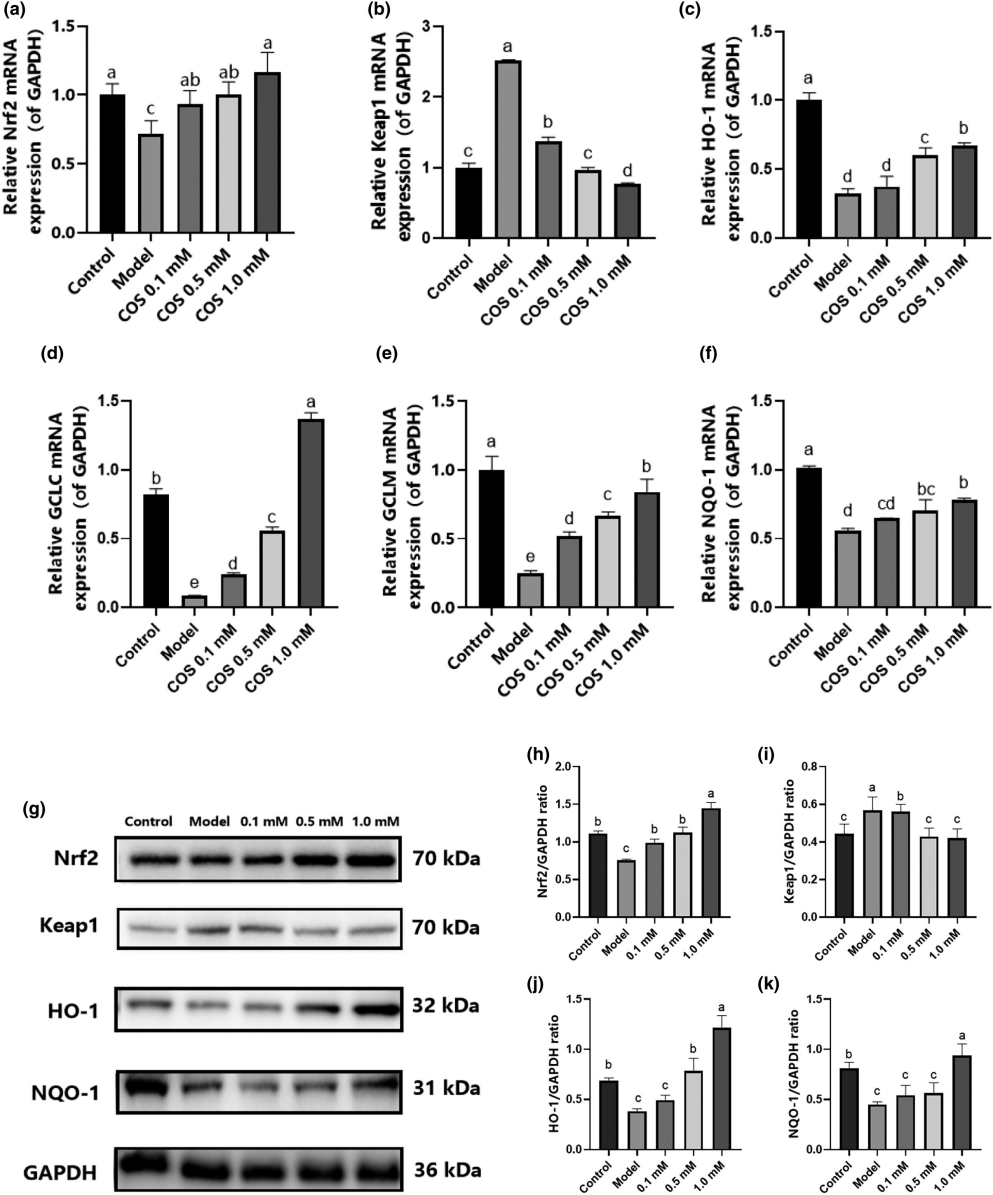
The effect of COS on the Nrf2/Keap1/ARE pathway in high glucose‐damaged HK‐2 cells. The mRNA expression levels of: (a) Nrf2. (b) Keap1. (c) HO‐1. (d) GCLC. (e) GCLM. (f) NQO‐1. (g) The Western blotting representations of the related factors, (h) Nrf2, (i) Keap1, (j) HO‐1, and (k) NQO‐1. Significant differences (*p* < .05) are indicated by different letters.

The effect of COS on the Nrf2/Keap1/ARE pathway was further verified in high glucose‐damaged HK‐2 cells. As illustrated in Figure 6, the *GCLM*, *GCLC*, *NQO‐1*, *HO‐1*, and *Nrf2* (Figure 6a,c–f) mRNA expression in the model group displayed a considerable decline (*p* < .05), while that in the pathway was reversed after COS intervention. In the Keap1 gene (Figure 6b), COS effectively reduced (*p* < .05) the *Keap1* mRNA expression, showing a dose‐dependent relationship. At the protein level (Figure 6g–k), the Keap1 level was considerably higher than in the normal group, while the Nrf2, HO‐1, and NQO‐1 levels were significantly lower (*p* < .05). COS treatment can reverse all these trends, which is consistent with the transcription level results. The findings indicated that COS intervention promoted Nrf2/Keap1 pathway expression.

## 4 DISCUSSION

Our previous research indicated that COS lowered blood sugar, improved blood lipid levels, and inhibited insulin cell apoptosis in a T2DM mouse model. However, whether COS can intervene in DN remains unclear (You et al., 2022). The current study showed that 12 weeks of COS intervention reduced the fasting blood glucose level and renal hypertrophy (*p* < .05) while stabilizing body weight. Furthermore, the UAER, UACR, BUN, and UA levels in the urine and the BUN, CRE, and UA in the serum of DN mice decreased significantly (*p* < .05). Moreover, COS reduced the mesangial matrix, prevented basement membrane thickening, and stabilized foot projection and the endothelial cell structure. These results indicated that COS alleviated renal injury and protected renal function.

Oxidative stress (OS) involves the production of a large number of ROS due to internal and external stimuli, which exceeds the original clearance capacity of the body and fails to achieve a balance between oxidation and the antioxidant system, resulting in pathological damage (Jha et al., 2016). Studies have shown that OS is the main cause of DN occurrence and progression (Bahreini et al., 2021; Dawood et al., 2022). Nrf2 primarily decreases OS via the transcription of various proteins (Ito et al., 2020; Shi et al., 2019) while regulating antioxidant enzyme expression (HO‐1 and NQO‐1) and second‐stage detoxification enzymes (GSH S‐transferase) via the ARE sequence composition of target genes (Das & Ghosh, 2017). In normal physiological conditions, Nrf2 forms a complex with Keap1 and E3 in the cytoplasm, which is degraded by ubiquitination to stabilize the concentration. However, when cells are stimulated by ROS, the cysteine residues on Keap1 are oxidized, resulting in Nrf2 dissociation from Keap1 and rapid transfer into the nucleus (Khaleel et al., 2019). The Nrf2 entering the nucleus binds to ARE to initiate downstream antioxidant enzyme transcription (Khaleel et al., 2019; Ruiz et al., 2013). COS upregulated the Nrf2, as well as downstream target genes (*GCLM*, *GCLC*, *NQO‐1*, and *HO‐1*) and proteins (HO‐1 and NQO‐1) in DN mice and HK‐2 cells with high‐glucose injury, and downregulated Keap1 gene and protein expression. Moreover, the docking results indicated that six hydrogen bond formation processes (Gly364, Arg415, Arg483, His436, Ser431, and Arg380) were involved in COS and Keap1 protein binding, demonstrating binding stability. It is speculated that COS may bind directly to Keap1, promote Nrf2 monomer entry into the nucleus, bind to antioxidant reaction elements, and upregulate downstream target gene expression. Therefore, the Nrf2/Keap1/ARE signaling pathway plays a role in the COS intervention mechanism.

## 5 CONCLUSION

The study findings indicate that COS intervention in mice with DN induced by a combination of high lipid levels and STZ can significantly reduce typical renal indexes, decrease OS indexes, and promote the Nrf2/Keap1/ARE pathway. In addition, high glucose‐damaged HK‐2 cell models confirm that COS can achieve an antioxidant effect via the Nrf2/Keap1/ARE mechanism. These findings may provide a scientific basis for practical COS application in developing food for special medical DN purposes.

## AUTHOR CONTRIBUTIONS

**Yuwen Liu:** Data curation (lead); formal analysis (lead); investigation (lead); methodology (lead); software (lead); validation (lead); visualization (lead); writing – original draft (lead). **Ran Zhang:** Investigation (lead); validation (lead); visualization (lead); writing – original draft (lead). **Jiaqi Zou:** Investigation (supporting); validation (supporting). **Hao Yin:** Methodology (supporting); resources (supporting). **Mengyao Zhao:** Investigation (lead); methodology (lead); writing – review and editing (lead). **Liming Zhao:** Conceptualization (lead); funding acquisition (lead); project administration (lead); resources (lead); supervision (lead); writing – review and editing (lead).

## CONFLICT OF INTEREST STATEMENT

There is no conflict of interest among authors.

## Supporting information


Data S1.

## Data Availability

The data used to support the results of this study are available upon request from the corresponding author.
