# Supplementary material for: The impact of chitooligosaccharides with a certain degree of polymerization on diabetic nephropathic mice and high glucose‐damaged HK‐2 cells
Source: Food Sci Nutr. 2024 Mar 22;12(6):4173–84. doi: 10.1002/fsn3.4078 (PMC11167136; doi:10.1002/fsn3.4078)
Supplement: Supplementary file 1 — Data S1. [file FSN3-12-4173-s001.docx]

**Supporting Information**

**COS Characterization**

Electrospray ionization source mass spectrometry (ESI-MS) was used to examine the COS mass. The results are presented in S.Fig. 1, showing a COS m/z of 663.2932, which corresponds to its [M+H^+^] ion peak.

As shown in S.Fig. 2, high-performance liquid chromatography (HPLC) was used to analyze the COS content. The results revealed a COS purity over 90%, while the COS deacetylation exceeded 95%.


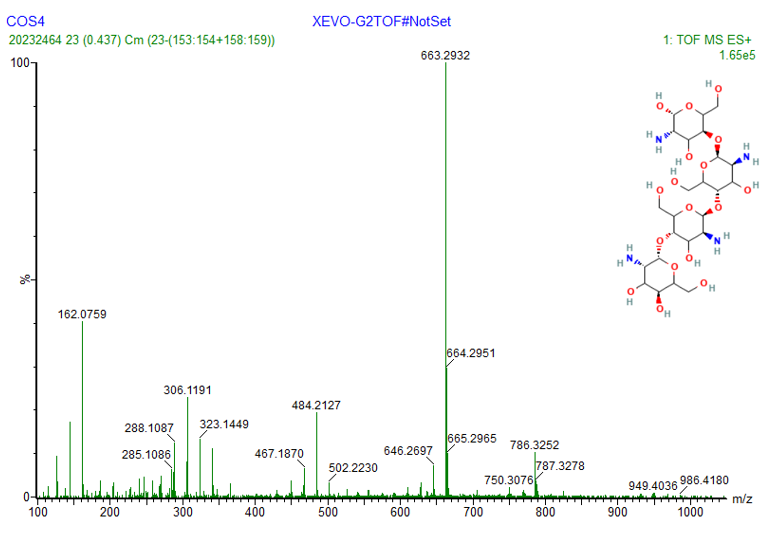


Supplementary Figure 1. The electrospray ionization source mass spectra of the COS.


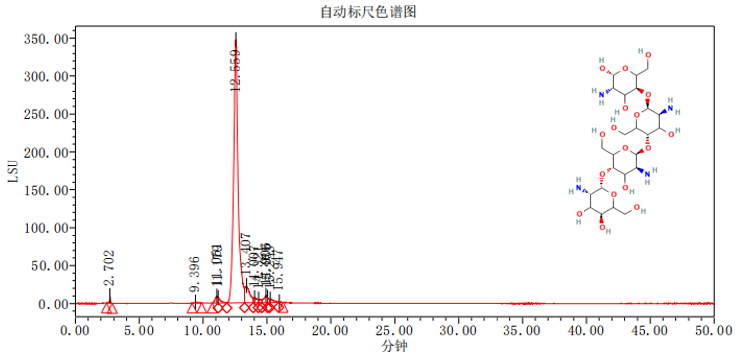


Supplementary Figure 2. The HPLC image of the COS (the horizontal coordinate is min, and the vertical coordinate is LSU).
